# Analysis of the inhibitors of apoptosis identifies BIRC3 as a facilitator of malignant progression in glioma

**DOI:** 10.18632/oncotarget.8657

**Published:** 2016-04-08

**Authors:** Loyola V. Gressot, Tiffany Doucette, Yuhui Yang, Gregory N. Fuller, Ganiraju Manyam, Arvind Rao, Khatri Latha, Ganesh Rao

**Affiliations:** ^1^ Department of Neurosurgery, The University of Texas MD Anderson Cancer Center, Houston, Texas 77030, USA; ^2^ Department of Pathology, The University of Texas MD Anderson Cancer Center, Houston, Texas 77030, USA; ^3^ Department of Bioinformatics and Computational Biology, The University of Texas MD Anderson Cancer Center, Houston, Texas 77030, USA

**Keywords:** inhibitor of apoptosis, BIRC3, glioma, malignant progression

## Abstract

Gliomas, the most common primary brain tumor in humans, include a spectrum of disease. High-grade gliomas (HGG), such as glioblastoma, may arise from low-grade gliomas (LGG) that have a more indolent course. The process of malignant transformation (MT) of LGG to HGG is poorly understood but likely involves the activation of signaling programs that suppress apoptosis. We previously showed that Survivin (BIRC5) plays a role in malignant progression of glioma. Here, we investigated the role of the remaining members of the Inhibitors of Apoptosis (IAP) family on promoting MT in glioma. Utilizing expression data from the cancer genome atlas (TCGA), we identified BIRC3 as a key facilitator of MT from LGG to HGG. TCGA HGGs with high expression of BIRC 3 demonstrated a survival disadvantage and expression levels of BIRC3 were also significantly higher in TCGA HGG compared to TCGA LGG cases. We validated our findings from TCGA by using matched human specimens to show that BIRC expression is increased in HGG compared to their precursor LGG lesions. Using a unique murine model of glioma, we show that overexpression of BIRC3 promotes higher grade glioma and significantly reduces tumor-free survival in mice.

## INTRODUCTION

Glioblastoma (GB), the most common primary malignant brain tumor in humans has a 5-year survival rate of only 10% despite aggressive treatment [1]. Secondary GBs are the result of an accumulation of molecular alterations and originate from low-grade gliomas (LGGs) [2]. LGGs often have an indolent clinical course until they progress to high-grade gliomas (HGGs) such as GB, after which survival time decreases significantly. The causes of this malignant progression are largely unknown.

We have shown previously that suppression of apoptosis can promote malignant progression in a murine model of glioma. Increased expression of Survivin (BIRC5), a member of the Inhibitors of Apoptosis (IAP) family, has been shown to be prognostic of shorter survival in patients with HGG [3, 4]. We showed that the overexpression of anti-apoptotic Survivin transcript variant 2 is associated with a poor prognosis as well as increased angiogenesis in a murine model [5]. While Survivin has been well studied, it is unknown if other IAPs are capable of inducing similar malignant degeneration. IAPs are a family of eight anti-apoptotic proteins whose overexpression has been linked to numerous malignancies including gliomas [6]. This family of 8 human proteins is characterized by a conserved baculoviral IAP repeat (BIR). IAP proteins modulate apoptosis directly binding caspases, inhibiting the assembly of pro-apoptotic molecules and promoting anti-apoptotic factors. Although multiple IAPs are known to be over expressed in gliomas, the specific role served by IAPs in the development and progression of gliomas remains unclear. Elucidating factors that facilitate the progression of gliomas and clarifying the role that IAPs play in this process is critical as mitigating their activity offers an opportunity for therapeutic intervention.

Here, we analyzed the Cancer Genome Atlas [7] to evaluate the differential expression of IAP family genes (save BIRC5 which we have studied previously) in LGG and HGG. We compared expression of these genes between LGG and HGG. We also evaluated the impact of IAP gene expression on survival. Of these, BIRC 3 (cIAP2) demonstrated increased expression in HGG relative to LGG. Increased expression of BIRC3 in human tumors (both HGG and LGG) also correlated with shorter survival. Based upon these findings, BIRC3 was determined to be a likely candidate to promote malignant progression from LGG to HGG. Expression of BIRC3 in a PDGFB-dependent murine model of glioma resulted in shorter-symptom free survival compared to controls and also resulted in a shift to a more malignant glioma phenotype. Thus, BIRC3 may be a potential therapeutic target to mitigate the transition from low-grade to high-grade glioma.

## RESULTS

### BIRC3 is highly differentially expressed in HGG compared to LGG

We compared mean RNA expression of individual IAPs (BIRC1-8) between LGG and GB tumors from TCGA data. Of these, BIRC3 and BIRC4 (XIAP) demonstrated the most significant differential log fold change of all the IAPs evaluated (Supplementary Table S1).

### BIRC3 expression is associated with shorter survival in LGG and GB patients

**Figure 1:**
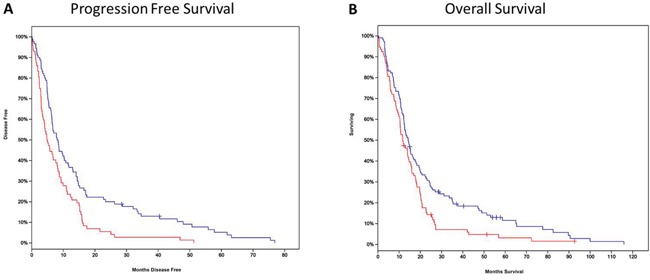
**A**. Progression-free survival (PFS) in patients from TCGA stratified by BIRC3 mRNA expression. The red line indicates patients with increased expression of BIRC3, and the blue line represents patients with decreased or non-expression relative to a normalized mean. PFS in expressors was significantly worse than the comparison group (log rank test, p= 0.00086). **B**. Overall survival (OS) in patients from TCGA stratified by BIRC3 mRNA expression. OS in expressors was significantly worse than the comparison group (log rank test, p= 0.009).

In low grade gliomas, median survival for BIRC3 overexpressors (N=153) was 26.7 months compared to 94.5 months for nonexpressors (N=364) (log rank test p= 0.009). BIRC2 overexpressors (N=249) also demonstrated a survival disadvantage (41.1 months versus 105.1 months for nonexpressors (N=271)) (Supplementary Table S2). However, in GB patients only BIRC3 overexpressors demonstrated a progression-free (PFS) and overall survival (OS) disadvantage compared to nonexpressors. PFS was 4.8 months for overexpressors (N=72) and 8.02 months for nonexpressors (N= 90) (log rank test, p=0.0009). OS was 11.7 months for overexpressors (N-93) compared to 14.3 months in nonexpressors (N=113) (log rank test p=0.01) (Figure 1A and 1B). For all other patients with increased expression of a particular IAP (from BIRC1 through BIRC8, excluding BIRC5, which we reported previously), median survival between expressors and non expressors was not statistically significantly different for GB TCGA cases (Supplementary Table S3).

### BIRC3 expression is upregulated in human HGG compared to LGG precursors

**Figure 2:**
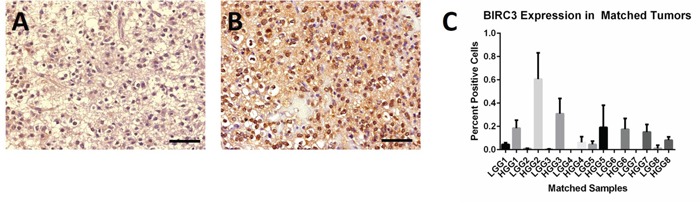
Expression of BIRC3 in representative human low-grade glioma and subsequent high-grade glioma **A**. Low-grade glioma demonstrating a paucity of BIRC3 staining. **B**. Subsequent high-grade glioma in the same patient demonstrating abundant BIRC3 staining (400X, scale bar= 50 μm). **C**. Bar graph demonstrating differences in BIRC3 staining in paired matched samples of LGG and HGG from eight patients.

Based on the result that increased BIRC3 expression was associated with shorter survival *and* increased expression in HGG compared to LGG, we wanted to know if it was upregulated in HGGs that progressed from previously diagnosed LGGs. We quantified the expression of BIRC 3 in human tumors and demonstrated an increase in expression between a panel of LGG (N=8) that eventually progressed to HGG (Figure 2). In 7/8 paired samples, we showed an increase in the number of tumor cells expressing BIRC3 in the HGG tumor compared to the matched LGG (one paired sample showed an increase in BIRC3 expression in the HGG relative to the low-grade precursor but the difference was not statistically significant) (Supplementary Table S4).

### BIRC3 overexpression promotes malignant progression *in vivo*

**Figure 3:**
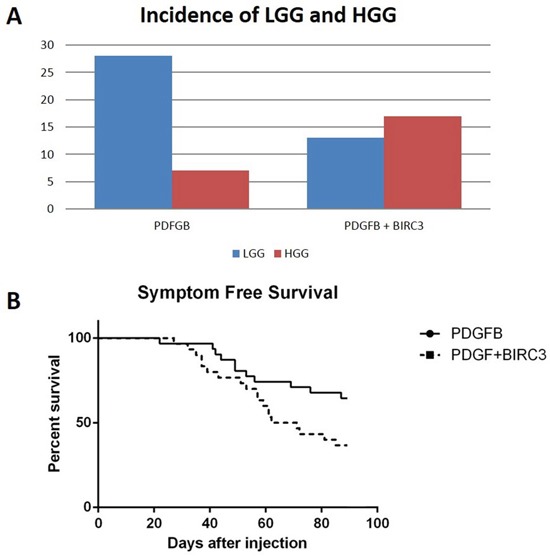
**A**. Incidence of low- and high-grade gliomas in the RCAS-PDGFB and RCAS-PDGFB + RCAS-BIRC3 injection sets. **B**. Kaplan-Meier curve demonstrating symptom-free survival in Ntv-a mice injected with RCAS-PDGFB and RCAS-PDGFB+RCAS-BIRC3.

**Figure 4:**
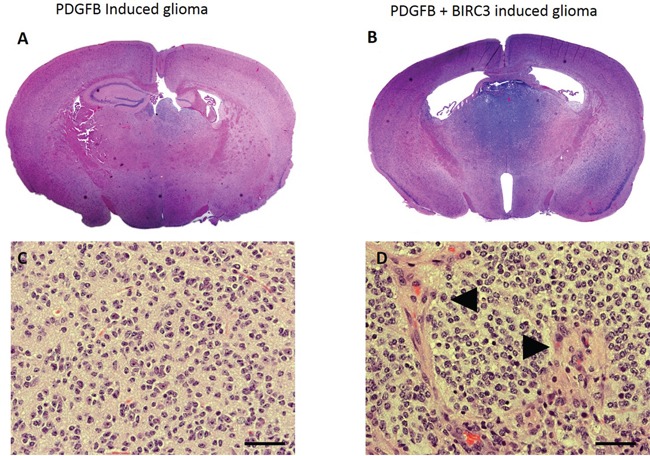
**A**. Whole mount photomicrograph of low-grade glioma induced by RCAS-PDGFB in Ntv-a mice. This is the dominant phenotype observed in this injection set. **B**. Whole mount photomicrograph of high-grade glioma with microvascular proliferation and necrosis induced by RCAS-PDGFB + RCAS-BIRC3 in Ntv-a mice. This is the dominant phenotype observed in this injection set. **C**. Magnified photomicrograph of the low-grade glioma induced by PDGFB demonstrating tumor cells without high-grade features. This is the dominant phenotype observed in this injection set. **D**. Magnified photomicrograph of high-grade glioma induced by PDGFB+BIRC3 demonstrating increased cellularity and areas of microvascular proliferation (arrowheads) consistent with HGG (400x, scale bar = 50 μm).

We interpreted the result that BIRC3 demonstrated a survival disadvantage in the TCGA LGG group as an indicator that it may promote malignant progression to HGG. The increased expression in GB compared to LGG also suggested its role in malignant transformation. We modeled BIRC3 overexpression using the RCAS/Ntv-a system. This is a genetically-engineered murine model of PDGFB-driven gliomas in which the low-grade phenotype is predominant. We overexpressed PDGFB alone or in conjunction with BIRC3 in an *in vivo* mouse model as well as BIRC3 independently. The mouse brains were harvested and evaluated for tumor formation and tumor grade (BIRC 3 expression was verified in the tumors, Supplementary Figure S1). The combination injection sets were compared to the rates of tumor formation for the PDGFB alone cohort (Figure 3A). In the PDGFB alone group, 31 out of 34 mice formed tumors (91%). Of these tumors, 22/31 were low grade (71%) 9/34 were high grade (29%). For the PDGFB + BIRC3 cohort, 30/34 mice formed tumors (88%) of which 13/30 were low grade (43%) and 17/30 were high grade (57%) (Figure 4). There were statistically more high-grade tumors in the combination BIRC3 + PDGFB cohort compared to PDGFB alone (chi square p=0.034). None of the mice injected with BIRC3 alone developed tumors.

### Increased IAP expression is associated with shorter tumor latency and survival *in vivo*

Mice were sacrificed when they developed neurological symptoms from tumor burden or at 90 days. For the PDGFB alone group, the median survival reached 90 days. In the combination cohort of PDGFB + BIRC3, the median survival was 67 days which was significantly shorter than the PDGFB alone cohort (log rank test p=0.036) (Figure 3B).

### BIRC3 induces apoptotic suppression in tumors

**Figure 5:**
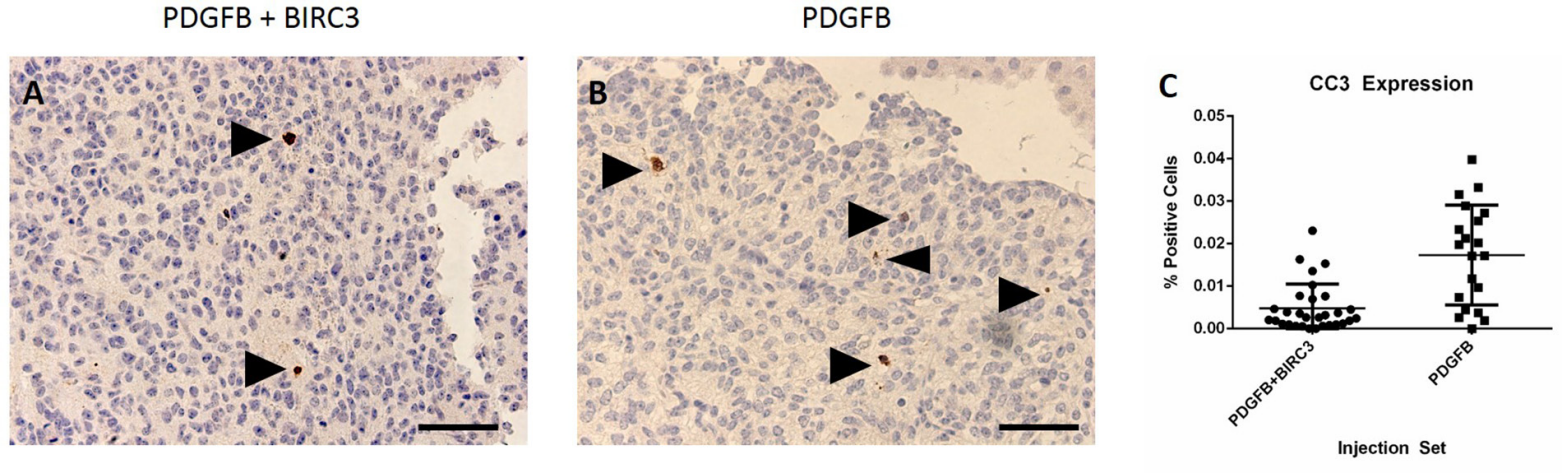
Suppression of apoptosis Decreased expression of cleaved caspase 3 was observed in tumors induced by RCAS-PDGFB + RCAS-BIRC3 relative to those induced by RCAS-PDGFB alone. **A**. CC3 staining in tumor induced by RCAS-PDGFB. **B**. CC3 staining in tumor induced by RCAS-PDGFB + RCAS-BIRC3. Arrowheads indicate positively staining apoptotic bodies. **C**. Scatter plot demonstrating difference in CC3 expression between injection sets (400x, scale bar= 50 μm).

We determined the extent of apoptotic suppression by comparing the percentage of CC3 positive cells in tumors induced from the RCAS-BIRC3 + RCAS-PDGFB and RCAS-PDGFB injection sets (Figure 5). For the RCAS-BIRC3 + RCAS-PDGFB injection set, the mean percentage of CC3 positive cells was 0.5% (± 0.1%). For the RCAS-PDGFB injection set, the mean percentage of CC3 positive cells was 1.7% (± 0.2%). The difference was statistically significant (unpaired t test, p<0.0001).

### Mitotic activity

**Figure 6:**
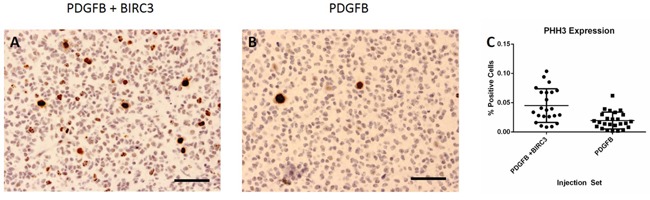
Tumor cell proliferation Increased expression of pHH3 was observed in tumors induced by RCAS-PDGFB + RCAS-BIRC3 relative to those induced by RCAS-PDGFB alone. **A**. pHH3 staining in tumor induced by RCAS-PDGFB. **B**. pHH3 staining in tumor induced by RCAS-PDGFB + RCAS-BIRC3. **C**. Scatter plot demonstrating difference in pHH3 expression between injection sets (400x, scale bar=50 μm).

We determined the extent of tumor cell proliferation by comparing the percentage of pHH3 positive cells in tumors induced from the RCAS-BIRC3 + RCAS-PDGFB and RCAS-PDGFB injection sets (Figure 6). For the RCAS-BIRC3 + RCAS-PDGFB injection set, the mean percentage of pHH3 positive cells was 4.5% (± 0.5%). For the RCAS-PDGFB injection set, the mean percentage of pHH3 positive cells was 2.0% (± 0.2%). The difference was statistically significant (unpaired t test, p=0.003).

## DISCUSSION

Evasion of apoptosis is a key pro-survival mechanism utilized by cancer cells to proliferate. The expression of *Survivin*, the best-studied member of the inhibitor of apoptosis family, has been shown to be a negative prognostic indicator in a variety of human cancers as well as gliomas [8, 9]. We showed recently that a transcript variant of *Survivin* has a profound effect on malignant progression in glioma and facilitated tumor cell proliferation [5]. In contrast, relatively little is known of the role played by the lesser-studied members of the IAP family in the development and progression of gliomas. Here, we studied IAP expression in the context of malignant progression using the TCGA dataset. We show that of the 8 known IAPs (excluding Survivin) BIRC3 has a unique role in facilitating glioma progression from low- to high-grade.

In an examination of TCGA expression data, we found that of all described IAPs, only BIRC3 both correlated with shorter survival in both LGG and GB and exhibited increased expression in GB relative to LGGs. Although a limitation of our study is that the TCGA data sets used were not matched (i.e. the HGG cases are not secondary GBs that arose from LGG precursors), the results from several hundred specimens with mRNA data proved useful in determining differences in expression between these tumor types. Further, a matched set of human LGGs that had eventually transformed to HGGs demonstrated increased expression of BIRC3 in the HGGs with essentially absent expression in the LGGs. These results prompted us to investigate the effect of BIRC3 overexpression *in vivo* using a genetically engineered murine model of endogenous brain tumor formation. Ectopic expression of PDGFB in Ntv-a mice induces gliomas of predominantly a low-grade phenotype. Combining PDGFB and BIRC3 resulted in a significant shift to a high-grade phenotype in the mice compared to PDGFB controls. Further, the combination of PDGFB and BIRC3 resulted in a significant decrease in tumor-free survival compared to controls. We did not observe any tumors in mice injected with BIRC3 alone indicating that it is insufficient to induce tumors independently.

A significant anti-apoptotic effect was observed in tumors induced with PDGFB and BIRC3 compared to PDGFB alone. This correlated inversely with an increase in tumor cell proliferation (as measured by mitotic activity). The uncoupling of apoptosis from proliferation has been described before in murine models of brain tumors [10, 11]. Similar to the present results, in these models the gene supplying the anti-apoptotic effect is insufficient to induce tumors independently. This is unsurprising as the drivers of the described GB subclasses do not generally include the increased expression of anti-apoptotic genes [12]. However, recently described multinucleated and giant cells that arise after radiation of GB demonstrate increased expression of BIRC3 among other prosurvival signals underscoring the importance of BIRC3 in the biology of high-grade glioma [13].

BIRC3 is a known target of the transcription factor NF-κB, which is associated with poor prognosis and apoptotic resistance in gliomas [14–16]. NF-κB is constitutively activated in HGG and target genes including BIRC3 (and BIRC5) are elevated in HGG compared to LGG [17]. In glioma cell lines with inhibited NF-κB expression, BIRC3 expression is also inhibited and these cells are sensitive to TNF-α induced cell death [17]. Thus, the expression of BIRC3 induced by NF-κB is necessary to inhibit TNF-α induced apoptosis. Interestingly, the activation of NF-κB is also induced by the ubiquitin-associated domain of cIAP2, and ubiquitin binding may facilitate cell survival, NF-κB signaling and subsequent oncogenesis [18]. Finally, hypoxia is a known stimulus for NF-κB and also induces BIRC3 in HGG [19]. As a hallmark of HGG, we frequently observe areas of necrosis – zones of hypoxia - in our high-grade murine tumors, and it is possible necrosis plays an additional stabilizing role in the expression of BIRC3.

XIAP (BIRC4) stabilizes BIRC3 thereby enhancing its expression [20]. Although in our analysis of TCGA data we found that increased expression of XIAP was seen in GB relative to LGG, this did not correlate with shorter survival. BIRC3 is fairly rapidly auto-ubiquinated and degraded but this process is inhibited by XIAP, at least in part by inhibiting ubiquitination. Because of the integration of numerous RCAS-BIRC3 sequences throughout Nestin+ progenitor cells, it is likely that there is at least a transient effect of BIRC3 expression prior to ubiquitination that yields a biological effect. A future direction for us is to express XIAP independently or in conjunction with BIRC3 to determine if a stabilized complex can induce a more malignant phenotype than either IAP independently. Importantly, the overexpression of XIAP independently is insufficient to stabilize endogenous BIRC3 implying that increased expression of BIRC3 must also occur to facilitate tumor growth and progression.

IAPs, in particular BIRC5, have been described as therapeutic targets in the treatment of cancer [21]. Down regulation of PI3K/AKT signaling using the IAP inhibitor, GDC-0152, which targets BIRC2, BIRC3, and XIAP has been shown to result in the induction of apoptosis in human leukemia cells [22]. Specific inhibition of BIRC3 has recently emerged as a treatment for the treatment of cancers including oral squamous cell carcinoma, colorectal cancers, and other malignancies [23, 24]. Strategies described include small molecule inhibitors, Smac (second mitochondria derived activator of caspase) mimetics that potentiate apoptosis and antagonize IAP proteins, and antisense oligonucleotides [23, 25–29]. Smac mimetics that antagonize BIRC3 activity have also demonstrated activity against melanoma cell lines when combined with TNF-α [30]. Although these treatments have yet to be attempted in brain tumors, our results demonstrating BIRC3 as upregulated in HGG relative to LGG and its effect on promoting malignant progression *in vivo* highlights its potential as a therapeutic target in glioma. Patients with the low-grade phenotype may survive for years (if not decades) but after malignant transformation, their survival declines precipitously and is measured in merely months thus mitigating malignant transformation from LGG to HGG is an appealing therapeutic strategy.

## MATERIALS AND METHODS

### Expression analysis

We determined mRNA expression levels in LGG and GB patients using the TCGA data portal (www.cbioportal.org) [31]. We defined overexpression if mRNA expression levels were higher than the normalized mean (using the oncoquery syntax described in cbioportal). The remaining patients were referred to as nonexpressors. The 2008 TCGA data set was used for the analysis because it contained the largest number of tumors with complete mRNA expression data. Analysis of TCGA data using this portal was performed between January and February of 2015.

### Gene expression analysis

Level 3 RNA sequencing data from the TCGA LGG (N=530) and GB (N=607) samples was used to quantify the expression of IAPs [32, 33]. Univariate analysis was performed on log2 transformed RNAseq data to identify differentially expressed IAPs between LGG and HGG using Student's t-test. The P-values obtained by multiple t-tests were adjusted using the Bonferroni method. Differentially expressed genes were defined as statistically significant if the Bonferroni-adjusted p-value was less than 0.05 and log fold change of the corresponding gene greater than 0.5.

### RCAS vector construction

The creation of RCAS-platelet-derived growth factor subunit B (PDGFB) was described previously [34]. We created RCAS-BIRC3 by cloning a human BIRC3 cDNA (MGC cDNA from GE Dharmacon) into a Gateway-compatible RCAS vector. To generate Gateway-compatible entry vectors containing the BIRC3 gene, we isolated the BIRC3 cDNA using sequences required for directional cloning into the pENTR/D-TOPO vectors (Invitrogen). The forward primer 5′ and reverse primers were used to generate the blunt-ended PCR products. The Gateway LR recombination reaction between the entry vector and BIRC3-containing entry vector resulted in RCAS-BIRC3 which was verified by sequencing.

### Transfection of DF-1 cells

Immortalized DF-1 chicken fibroblasts were grown in Dulbecco's modified Eagle's medium containing 10% fetal bovine serum in a humidified atmosphere of 95% air/5% CO_2_ at 37 degrees C. Live virus was produced by transfecting the RCAS vector into DF-1 cells using Fugene (Roche) and allowing them to replicate in culture.

### Verification of BIRC3 expression in mouse and human tumors

We verified BIRC3 expression by immunofluorescence. Untransfected DF-1 cells were used as a negative control. DF-1 cells infected with RCAS-BIRC3 were then stained with a human-specific anti-BIRC3 antibody (Thermo Scientific). To verify expression of BIRC3 in tumor-bearing Ntv-a mice, we stained the brains with the anti-BIRC3 antibody and compared this to un-injected controls. A panel (N= 8) of human LGGs and matched HGGs to which they had progressed were also stained with the human specific anti-BIRC3 antibody. We counted the number of BIRC3 positive cells in five non-overlapping microscopic fields (400X magnification). Positive cells were compared between LGGs and HGGs. Acquisition of human tumors was approved by the institutional review board at the University of Texas MD Anderson Cancer Center (PA14-0709).

### *In vivo* somatic cell transfer in Ntv-a mice

The generation of transgenic Ntv-a mice, which are a mix of strains C57BL/6, BALB/c, FVB/N, and CD1, was previously described [35]. To transfer genes, DF-1 producer cells transfected with an RCAS vector (1 × 10^4^ cells in 1–2 μL of PBS) were injected bilaterally into the frontal lobes of mice with a 10-μL gas-tight Hamilton syringe. The mice were injected within 24–72 hours after birth during which the Nestin+ cells producing TVA receptors are most proliferative. Equal amounts of DF-1 cells were injected in the injection sets consisting of two RCAS vectors. We co-expressed BIRC3 and PDGFB and independently in Ntv-a mice. Consistent with our previous studies mice were humanely euthanized by carbon dioxide asphyxiation 90 days post-injection or sooner if symptoms related to tumor burden (e.g., hydrocephalus) were present [5, 11, 36]. The brains were removed, fixed in formalin, embedded in paraffin, and sectioned for immunohistochemical analysis. Hematoxylin and eosin staining allowed for analysis of tumor formation. The animal experiments performed in this research were approved by The Institutional Animal Care and Use Committee at The University of Texas MD Anderson Cancer Center (Protocol No. 00000900-RN01).

### Determination of tumor grade

Tumor grade was determined by a neuropathologist (GNF) using WHO 2007 criteria. Low-grade tumors were identified increased cellularity owing to infiltrating tumor cells. High-grade tumors were identified by the presence of microvascular proliferation, foci of necrosis, or brisk mitotic activity.

### Apoptotic assay

Apoptosis was detected and quantified in tumors from the different injection sets by immunostaining of formalin-fixed, paraffin-embedded tumor sections with an antibody against cleaved caspase-3 (CC3) (1:50, 5A1E, Cell Signaling Technology, Danvers, MA). We counted the total number of cells and the number of positively stained cells in the area of highest tumor cell density in five non-overlapping high-power microscopic fields (400x magnification). At least five tumors from each injection set were selected for analysis. The expression level was calculated as the percentage of positive cells in each field.

### Proliferation assay

To determine the extent of tumor cell proliferation in the different injection sets, we analyzed formalin-fixed, paraffin-embedded tumor-bearing tissue sections with an antibody against pHH3, which has been described as a useful marker for determining the mitotic index in gliomas [37]. We counted the total number of cells and the number of positively stained cells in the area of highest tumor cell density in 10 non-overlapping high-power microscopic fields (400× magnification) in tumor-bearing brains taken from 5 mice in each injection set. The mitotic index was calculated as the percentage of positive cells in each field.

### Survival statistics

The incidence of tumors between injection sets was assessed using the chi-square test with a p<0.05 being considered significant. Non-adjusted tumor latency was reported by Kaplan-Meier curves. The log rank test was used to compare survival curves with p<0.05 being considered significant. Statistical analysis was carried out using Graphpad Prism, version 6 software.

### Ethical statements

All applicable international, national, and/or institutional guidelines for the care and use of animals were followed. All procedures performed in studies involving animals were in accordance with the ethical standards of the institution or practice at which the studies were conducted.

All procedures performed in studies involving human participants were in accordance with the ethical standards of the institutional and/or national research committee and with the 1964 Helsinki declaration and its later amendments or comparable ethical standards.

## SUPPLEMENTARY MATERIALS FIGURES AND TABLES



## References

[1] Stupp R, Hegi ME, Mason WP, van den Bent MJ, Taphoorn MJ, Janzer RC, Ludwin SK, Allgeier A, Fisher B, Belanger K, Hau P, Brandes AA, Gijtenbeek J, Marosi C, Vecht CJ, Mokhtari K (2009). Effects of radiotherapy with concomitant and adjuvant temozolomide versus radiotherapy alone on survival in glioblastoma in a randomised phase III study: 5-year analysis of the EORTC-NCIC trial. Lancet Oncol.

[2] Huse JT, Phillips HS, Brennan CW (2011). Molecular subclassification of diffuse gliomas: seeing order in the chaos. Glia.

[3] Chakravarti A, Noll E, Black PM, Finkelstein DF, Finkelstein DM, Dyson NJ, Loeffler JS (2002). Quantitatively determined survivin expression levels are of prognostic value in human gliomas. J Clin Oncol.

[4] Huang Y, Chen X, Chen N, Nie L, Xu M, Zhou Q (2011). Expression and prognostic significance of survivin splice variants in diffusely infiltrating astrocytoma. J Clin Pathol.

[5] Doucette T, Latha K, Yang Y, Fuller GN, Rao A, Rao G (2014). Survivin transcript variant 2 drives angiogenesis and malignant progression in proneural gliomas. Neuro Oncol.

[6] Fulda S, Vucic D (2012). Targeting IAP proteins for therapeutic intervention in cancer. Nat Rev Drug Discov.

[7] The Cancer Genome Atlas

[8] Tanabe H, Yagihashi A, Tsuji N, Shijubo Y, Abe S, Watanabe N (2004). Expression of survivin mRNA and livin mRNA in non-small-cell lung cancer. Lung Cancer.

[9] Altieri DC (2003). Survivin, versatile modulation of cell division and apoptosis in cancer. Oncogene.

[10] McCall TD, Pedone CA, Fults DW (2007). Apoptosis suppression by somatic cell transfer of Bcl-2 promotes Sonic hedgehog-dependent medulloblastoma formation in mice. Cancer Res.

[11] Doucette T, Yang Y, Zhang W, Fuller GN, Suki D, Fults DW, Rao G (2011). Bcl-2 promotes malignant progression in a PDGF-B-dependent murine model of oligodendroglioma. Int J Cancer.

[12] Verhaak RG, Hoadley KA, Purdom E, Wang V, Qi Y, Wilkerson MD, Miller CR, Ding L, Golub T, Mesirov JP, Alexe G, Lawrence M, O'Kelly M, Tamayo P, Weir BA, Gabriel S (2010). Integrated genomic analysis identifies clinically relevant subtypes of glioblastoma characterized by abnormalities in PDGFRA, IDH1, EGFR, and NF1. Cancer Cell.

[13] Kaur E, Rajendra J, Jadhav S, Shridhar E, Goda JS, Moiyadi A, Dutt S (2015). Radiation-induced homotypic cell fusions of innately resistant glioblastoma cells mediate their sustained survival and recurrence. Carcinogenesis.

[14] Robe PA, Bentires-Alj M, Bonif M, Rogister B, Deprez M, Haddada H, Khac MT, Jolois O, Erkmen K, Merville MP, Black PM, Bours V (2004). *In vitro* and in vivo activity of the nuclear factor-kappaB inhibitor sulfasalazine in human glioblastomas. Clin Cancer Res.

[15] Korkolopoulou P, Levidou G, Saetta AA, El-Habr E, Eftichiadis C, Demenagas P, Thymara I, Xiromeritis K, Boviatsis E, Thomas-Tsagli E, Panayotidis I, Patsouris E (2008). Expression of nuclear factor-kappaB in human astrocytomas: relation to pI kappa Ba, vascular endothelial growth factor, Cox-2, microvascular characteristics, and survival. Hum Pathol.

[16] Angileri FF, Aguennouz M, Conti A, D La Torre, Cardali S, Crupi R, Tomasello C, Germano A, Vita G, Tomasello F (2008). Nuclear factor-kappaB activation and differential expression of survivin and Bcl-2 in human grade 2-4 astrocytomas. Cancer.

[17] Zhao X, Laver T, Hong SW, Twitty GB, Devos A, Devos M, Benveniste EN, Nozell SE (2011). An NF-kappaB p65-cIAP2 link is necessary for mediating resistance to TNF-alpha induced cell death in gliomas. J Neurooncol.

[18] Gyrd-Hansen M, Darding M, Miasari M, Santoro MM, Zender L, Xue W, Tenev T, da Fonseca PC, Zvelebil M, Bujnicki JM, Lowe S, Silke J, Meier P (2008). IAPs contain an evolutionarily conserved ubiquitin-binding domain that regulates NF-kappaB as well as cell survival and oncogenesis. Nature cell biology.

[19] Murat A, Migliavacca E, Hussain SF, Heimberger AB, Desbaillets I, Hamou MF, Ruegg C, Stupp R, Delorenzi M, Hegi ME (2009). Modulation of angiogenic and inflammatory response in glioblastoma by hypoxia. PLoS One.

[20] Yang W, Cooke M, Duckett CS, Yang X, Dorsey JF (2014). Distinctive effects of the cellular inhibitor of apoptosis protein c-IAP2 through stabilization by XIAP in glioblastoma multiforme cells. Cell Cycle.

[21] Owens TW, Gilmore AP, Streuli CH, Foster FM (2013). Inhibitor of Apoptosis Proteins: Promising Targets for Cancer Therapy. J Carcinog Mutagen.

[22] Hu R, Li J, Liu Z, Miao M, Yao K (2015). GDC-0152 induces apoptosis through down-regulation of IAPs in human leukemia cells and inhibition of PI3K/Akt signaling pathway. Tumour biology: the journal of the International Society for Oncodevelopmental Biology and Medicine.

[23] Miura K, Karasawa H, Sasaki I (2009). cIAP2 as a therapeutic target in colorectal cancer and other malignancies. Expert opinion on therapeutic targets.

[24] Nagata M, Nakayama H, Tanaka T, Yoshida R, Yoshitake Y, Fukuma D, Kawahara K, Nakagawa Y, Ota K, Hiraki A, Shinohara M (2011). Overexpression of cIAP2 contributes to 5-FU resistance and a poor prognosis in oral squamous cell carcinoma. British journal of cancer.

[25] Bertrand MJ, Milutinovic S, Dickson KM, Ho WC, Boudreault A, Durkin J, Gillard JW, Jaquith JB, Morris SJ, Barker PA (2008). cIAP1 and cIAP2 facilitate cancer cell survival by functioning as E3 ligases that promote RIP1 ubiquitination. Molecular cell.

[26] Galban S, Hwang C, Rumble JM, Oetjen KA, Wright CW, Boudreault A, Durkin J, Gillard JW, Jaquith JB, Morris SJ, Duckett CS (2009). Cytoprotective effects of IAPs revealed by a small molecule antagonist. Biochem J.

[27] LaCasse EC, Cherton-Horvat GG, Hewitt KE, Jerome LJ, Morris SJ, Kandimalla ER, Yu D, Wang H, Wang W, Zhang R, Agrawal S, Gillard JW, Durkin JP (2006). Preclinical characterization of AEG35156/GEM 640, a second-generation antisense oligonucleotide targeting X-linked inhibitor of apoptosis. Clin Cancer Res.

[28] Swinney DC, Xu YZ, Scarafia LE, Lee I, Mak AY, Gan QF, Ramesha CS, Mulkins MA, Dunn J, So OY, Biegel T, Dinh M, Volkel P, Barnett J, Dalrymple SA, Lee S (2002). A small molecule ubiquitination inhibitor blocks NF-kappa B-dependent cytokine expression in cells and rats. J Biol Chem.

[29] Wu G, Chai J, Suber TL, Wu JW, Du C, Wang X, Shi Y (2000). Structural basis of IAP recognition by Smac/DIABLO. Nature.

[30] Krepler C, Chunduru SK, Halloran MB, He X, Xiao M, Vultur A, Villanueva J, Mitsuuchi Y, Neiman EM, Benetatos C, Nathanson KL, Amaravadi RK, Pehamberger H, McKinlay M, Herlyn M (2013). The novel SMAC mimetic birinapant exhibits potent activity against human melanoma cells. Clin Cancer Res.

[31] Cerami E, Gao J, Dogrusoz U, Gross BE, Sumer SO, Aksoy BA, Jacobsen A, Byrne CJ, Heuer ML, Larsson E, Antipin Y, Reva B, Goldberg AP, Sander C, Schultz N (2012). The cBio cancer genomics portal: an open platform for exploring multidimensional cancer genomics data. Cancer Discov.

[32] (2008). Comprehensive genomic characterization defines human glioblastoma genes and core pathways. Nature.

[33] Brennan CW, Verhaak RG, McKenna A, Campos B, Noushmehr H, Salama SR, Zheng S, Chakravarty D, Sanborn JZ, Berman SH, Beroukhim R, Bernard B, Wu CJ, Genovese G, Shmulevich I, Barnholtz-Sloan J (2013). The somatic genomic landscape of glioblastoma. Cell.

[34] Dai C, Celestino JC, Okada Y, Louis DN, Fuller GN, Holland EC (2001). PDGF autocrine stimulation dedifferentiates cultured astrocytes and induces oligodendrogliomas and oligoastrocytomas from neural progenitors and astrocytes in vivo. Genes Dev.

[35] Holland EC, Varmus HE (1998). Basic fibroblast growth factor induces cell migration and proliferation after glia-specific gene transfer in mice. Proc Natl Acad Sci U S A.

[36] Gressot LV, Doucette TA, Yang Y, Fuller GN, Heimberger AB, Bogler O, Rao A, Latha K, Rao G (2014). Signal transducer and activator of transcription 5b drives malignant progression in a PDGFB-dependent proneural glioma model by suppressing apoptosis. Int J Cancer.

[37] Colman H, Giannini C, Huang L, Gonzalez J, Hess K, Bruner J, Fuller G, Langford L, Pelloski C, Aaron J, Burger P, Aldape K (2006). Assessment and prognostic significance of mitotic index using the mitosis marker phospho-histone H3 in low and intermediate-grade infiltrating astrocytomas. Am J Surg Pathol.

