# Supplementary material for: Analysis of the inhibitors of apoptosis identifies BIRC3 as a facilitator of malignant progression in glioma
Source: Oncotarget. 2016 Apr 8;8(8):12695–704. doi: 10.18632/oncotarget.8657 (PMC5355046; doi:10.18632/oncotarget.8657)
Supplement: Supplementary file 1 [file oncotarget-08-12695-s001.pdf]

SUPPLEMENTARY FIGURE AND TABLES

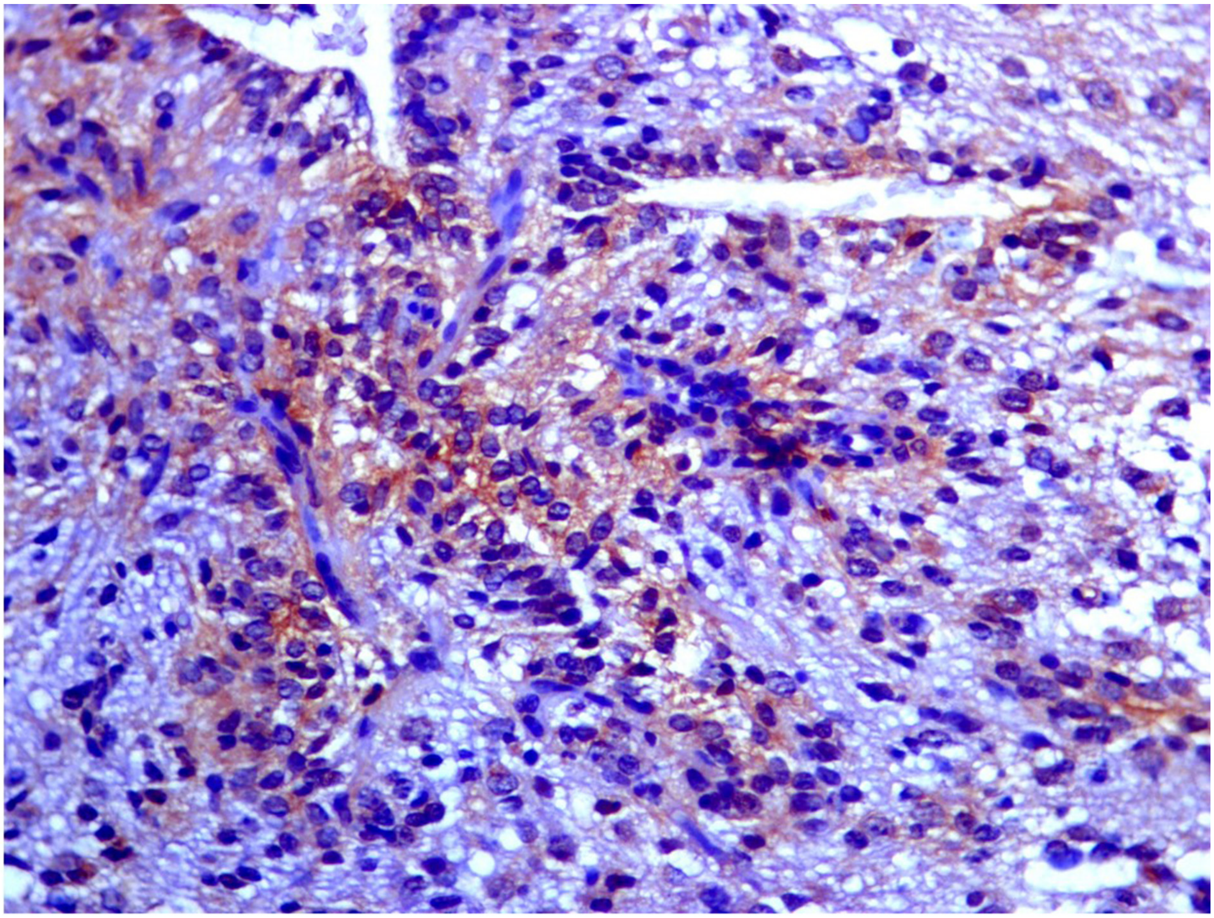

Supplementary Figure S1: BIRC3 staining in a HGG induced by PDGFB+BIRC3. 400X magnification.

Supplementary Table S1: Differential RNA expression levels of IAPs in LGG and GBM

Gene	LGG Expression (Mean)	GBM Expression (Mean)	Fold Change
BIRC1	0.000693555	0.001361643	-0.000668088
BIRC2	1.926767542	2.208344969	-0.281577427
BIRC3	1.000403772	1.548315026	-0.547911254
BIRC4	0.743276347	1.542989543	-0.799713197
BIRC5	0.124728973	0.002655731	0.122073242
BIRC6	0.009806117	0.010080691	-0.000274574
BIRC7	3.942221594	3.515346676	0.426874917
BIRC8	0.00452855	0.003870621	0.000657928

Supplementary Table S2: Comparison of median survival between expressors and non expressors of IAPs (excluding BIRC5) in TCGA Low-grade glioma patients

IAP	Median Survival Expressor (months)	Median Survival Non-expressor (months)	Log rank test p value
BIRC1	75.1	87.4	0.85
BIRC2	41.1	105.1	0.0000025
BIRC3	26.7	94.5	0.00018
BIRC4	105.12	78.2	0.33
BIRC6	N/A	87.4	0.35
BIRC7	26.74	87.4	0.71
BIRC8	133.6	94.5	0.71

Supplementary Table S3: Comparison of median survival between expressors and non expressors of IAPs (excluding BIRC5) in TCGA High-grade glioma patients

IAP	Median Survival Expressor (months)	Median Survival Non-expressor (months)	Log rank test p value
BIRC1	14.3	13	0.37
BIRC2	12.2	14.2	0.07
BIRC3	11.7	14.3	0.01
BIRC4	14.3	12.9	0.37
BIRC6	12.6	14	0.38
BIRC7	12.6	14.5	0.27
BIRC8	14.1	11.7	0.42

Supplementary Table S4: Difference in BIRC3 expression between matched LGG and HGG samples based on the number of positive tumor cells in 5 non-overlapping fields from each tumor

Paired Sample	LGG	HGG	T Test P value
1	0.04481 ± 0.006423	0.1842 ± 0.03067	0.0021
2	0.007794 ± 0.001970	0.6065 ± 0.1010	0.0004
3	0.001950 ± 0.001950	0.3090 ± 0.06580	0.0011
4	0.0 ± 0.0	0.06264 ± 0.02174	0.0205
5	0.04546 ± 0.01251	0.1910 ± 0.08549	0.1305
6	0.0 ± 0.0	0.1756 ± 0.04122	0.0028
7	0.0 ± 0.0	0.1508 ± 0.02978	0.0010
8	0.01164 ± 0.01164	0.08244 ± 0.01392	0.0056
